# Glasgow Coma Scale score at intensive care unit discharge predicts the 1-year outcome of patients with severe traumatic brain injury

**DOI:** 10.1007/s00068-013-0269-3

**Published:** 2013-03-05

**Authors:** J. Leitgeb, W. Mauritz, A. Brazinova, M. Majdan, I. Janciak, I. Wilbacher, M. Rusnak

**Affiliations:** 1 Department of Traumatology, Medical University of Vienna, Währinger Gürtel 18–20, 1090 Vienna, Austria; 2 Anaesthesiology and Intensive Care Medicine, Trauma Hospital “Lorenz Boehler”, Vienna, Austria; 3 International Neurotrauma Research Organization (INRO), Vienna, Austria; 4 Department of Public Health, Faculty of Health and Social Services, Trnava University, Trnava, Slovak Republic

**Keywords:** Traumatic brain injury, Severe, Glasgow Coma Scale score, Glasgow Outcome Scale score, Long-term outcomes

## Abstract

**Objective:**

To analyse the association between the Glasgow Coma Scale (GCS) score at intensive care unit (ICU) discharge and the 1-year outcome of patients with severe traumatic brain injury (TBI).

**Design:**

Retrospective analysis of prospectively collected observational data.

**Patients:**

Between 01/2001 and 12/2005, 13 European centres enrolled 1,172 patients with severe TBI. Data on accident, treatment and outcomes were collected. According to the GCS score at ICU discharge, survivors were classified into four groups: GCS scores 3–6, 7–9, 10–12 and 13–15. Using the Glasgow Outcome Scale (GOS), 1-year outcomes were classified as “favourable” (scores 5, 4) or “unfavourable” (scores <4). Factors that may have contributed to outcomes were compared between groups and for favourable versus unfavourable outcomes within each group.

**Main results:**

Of the 538 patients analysed, 308 (57 %) had GCS scores 13–15, 101 (19 %) had scores 10–12, 46 (9 %) had scores 7–9 and 83 (15 %) had scores 3–6 at ICU discharge. Factors significantly associated with these GCS scores included age, severity of trauma, neurological status (GCS, pupils) at admission and patency of the basal cisterns on the first computed tomography (CT) scan. Favourable outcome was achieved in 74 % of all patients; the rates were significantly different between GCS groups (93, 83, 37 and 10 %, respectively). Within each of the GCS groups, significant differences regarding age and trauma severity were found between patients with favourable versus unfavourable outcomes; neurological status at admission and CT findings were not relevant.

**Conclusion:**

The GCS score at ICU discharge is a good predictor of 1-year outcome. Patients with a GCS score <10 at ICU discharge have a poor chance of favourable outcome.

## Introduction

During the last 10 years, the International Neurotrauma Research Organization (INRO; based in Vienna, Austria, founded in 1999) has coordinated a number of projects which focused on the treatment of patients with severe traumatic brain injury (TBI). An EU-funded project implemented TBI guidelines and evaluated the effects on outcomes in centres from Bosnia, Croatia and Macedonia [1]. An Austrian project analysed the epidemiology [2], treatment [3–5] and effects of guideline-based treatment of patients with severe TBI [6]. Smaller projects studied regional variations of TBI treatment and outcomes in Slovakia. A database developed by the INRO was used to collect the data for these projects; today, this database has data on 1,172 patients with severe TBI. As all these projects were purely observational, this database includes data on paediatric as well as geriatric patients, and on patients with low Glasgow Coma Scale (GCS) scores.

A number of studies have focused on the prediction of outcomes of patients with severe TBI [7, 8], and the proposed scores are useful for predicting unfavourable outcome or death using variables available at hospital admission. Patients with severe TBI are usually admitted to intensive care units (ICUs) and between 35 and 50 % of these patients die in the ICU [9, 10]. Many of the ICU survivors are still in a somehow compromised neurological state; parameters that predict further outcomes for these patients would be welcomed by clinicians as well as relatives of the patients. Unfortunately, there are no predictive models for these patients. It has been shown that the 3-month GCS score shows good correlation with long-term outcomes [11] but this is far away in the future of these patients. Many clinicians assume that the GCS score at ICU discharge has some predictive value with regard to long-term outcome, although this has never been proven or even investigated. We, therefore, decided to carry out an analysis of the association between the GCS score at ICU discharge and long-term outcome using our database of severe TBI cases. Our hypothesis was that the GCS score at ICU discharge would predict the outcome at 12 months after trauma.

## Materials and methods

The data for this study were collected in 13 centres located in Austria, Bosnia, Croatia, Macedonia and Slovakia. All centres were of the tertiary-care level; treatment quality, however, was not uniform [1], as the centres from the lower income countries were less able to provide treatment according to the guidelines for TBI management [12]. The centres [possible Therapy Intensity Level (TIL) given in square brackets] included six University Departments of Neurosurgery (Graz [4], Osijek [4], Rijeka [4], Sarajevo [2], Skopje [1] and Zagreb [4]), five Departments of Neurosurgery at large City Hospitals (Banská Bystrica [3], Klagenfurt [4], Martin [2], Michalovce [2] and Salzburg [4]), one free-standing Centre for Neurosurgery and Neurology (Linz [4]) and one free-standing Trauma Centre (Vienna [4]). Treatment quality was significantly associated with long-term outcomes: compared to expected mortality rates, hospital mortality was 6.5 % lower in Austrian centres, 2.4 % lower in the Croatian and Slovak centres, and 13 % higher in Sarajevo and Skopje [1]. There was no “centre effect” for post-ICU mortality, i.e. the main differences between the centres were the possible TIL and/or quality of intensive care.

The data were collected between January 2001 and June 2005, but none of the centres provided data for more than 3 years. This reflects the fact that the data were collected for different projects. All projects were purely observational and prospectively enrolled all patients that were admitted to the study centres during their period of data collection. Patients were included if they had “severe TBI” according to the criteria defined by the US National Traumatic Coma Data Bank [13], such as a GCS score of 8 or less following resuscitation or a GCS score deteriorating to 8 or less within 48 h of injury. Only patients who survived at least until admission to the ICU were enrolled into this study.

Treatment in the field was provided by emergency physicians or paramedics. All patients had a rapid examination which included documentation of vital signs (GCS, pupillary status, blood pressure, heart rate, oxygen saturation). Rapid sequence intubation facilitated by hypnotics and relaxants, ventilation, treatment of haemorrhage and fluid resuscitation were done as appropriate. After admission, each patient was examined by a trauma team (anaesthesiologists, trauma surgeons or neurosurgeons, radiologists, nurses) and a computed tomography (CT) scan was performed as soon as possible. The patients then underwent surgery as appropriate and/or were admitted to the ICU. Surgical care was provided by neurosurgeons alone (Departments of Neurosurgery) or by trauma surgeons (Trauma Departments, Trauma Centre), who had the option of consulting neurosurgeons if deemed necessary. Intensive care was provided by anaesthesiologists in cooperation with neuro- or trauma surgeons. The whole treatment process in each centre was supposed to be based on the guidelines for the management of patients with severe TBI published by the Brain Trauma Foundation [12] and introduced at the start of the projects in each centre.

Data collection was done using the International Traumatic Coma Project (ITCP) database, which allowed for data collection over the Internet. Basic demographic data of the patient, cause and location of trauma, pre-hospital status and treatment, mechanism and severity of trauma, results of CT scans, results of laboratory testing and data on surgical procedures and outcomes were recorded. Pre-hospital data were documented by the local paramedics, and these data were then transferred into the ITCP database. CT scans were interpreted by neurosurgeons, trauma surgeons and radiologists, and the summarised findings were entered into a separate CT page in the ITCP database. This page collected detailed data on basal cisterns (open/closed), midline shift (in mm) and main findings [subdural haematoma (SDH), oedema, contusions etc.]. No central review of CT scans was done, as no actual images were uploaded into the ITCP database. Information on status and treatment was recorded in detail for the first 10 days. In addition, data on the duration of various treatments, on complications and on outcome were collected at discharge from the ICU. Information on status and location was recorded at 3, 6 and 12 months after injury. This was done by phone calls to the patients and/or their relatives; in some cases, the Glasgow Outcome Scale (GOS) score was recorded at patients’ follow-up visits to the centres. In most centres, data were collected by local research fellows; data quality was monitored by the INRO data manager. Missing or implausible data were reported to local research fellows, who then submitted missing or corrected values. In the Austrian centres, data were extracted from the prospectively collected records by a single researcher (I.W.), who visited the centres at regular intervals. Personal data protection was observed and the identifiers were kept separately from the data.

Files from all patients who survived their ICU stay were selected for this analysis. Data on trauma mechanism, trauma severity, CT findings, treatment and outcomes were retrieved for each patient. Files that did not include information about 1-year outcomes were excluded from further analysis. The Trauma and Injury Severity Score (TRISS) method [14] was used to estimate the probability of hospital survival (Ps). To describe 1-year outcomes, the GOS was used [15]. “Favourable outcome” was defined as a GOS score of 5 or 4 and “unfavourable outcome” was defined as a GOS score of 3 or less at 12 months after trauma. According to the GCS score at ICU discharge, the patients were classified into four groups: GCS scores 3–6, 7–9, 10–12 and 13–15. Differences between these four groups were analysed to identify the factors that contributed to the status at ICU discharge. To identify factors contributing to 1-year outcomes, within each of the four groups, patients who reached favourable outcomes were compared to those that had unfavourable outcomes.

Statistical analysis was performed with the open-source statistical package R. Chi-square tests or Fisher’s exact test were used, as appropriate, to test differences between proportions, two-way analysis of variance was used to test differences between means and the Kruskal–Wallis test was used to test differences between medians when comparing the four categories. For pairwise comparisons, the two-sample *t*-test was used for comparisons of means and the two-sample Wilcox test was used for comparisons of medians. A *p*-value of <0.05 was considered to be statistically significant. Factors that were significant in the univariate analysis were entered into logistic regression models, with backward elimination of non-significant factors. These factors included age (per year increase), trauma mechanism (“fall” vs. “other”), GCS, Injury Severity Score (ISS), Abbreviated Injury Scale (AIS) “head” score, basal cisterns (“open” vs. “compressed/closed”), midline shift (“<5 mm” vs.“>5 mm”) and pupillary status (“normal” vs. “abnormal”). Two models were constructed in order to elucidate which factors influence hospital survival and favourable outcome one year after trauma, respectively.

## Results

Of the 1,172 patients in the database, 423 (36 %) died in the ICU. In the remaining 749 survivors, neither GCS scores at ICU discharge nor GOS scores at 12 months were recorded in 70 cases (9 % of survivors); these had to be excluded. In 141 cases (19 % of survivors), GOS scores at 12 months after trauma were not available; of these, 81 (57 %) had a GCS score of 13–15 at ICU discharge, 37 (26 %) had a score of 10–12, 11 (8 %) had a score of 7–9 and 12 (9 %) had a score of 3–6. These proportions were similar to those found in the patients where both GCS at ICU discharge and GOS at 12 months after trauma had been recorded, and outcomes comparable to those reported below are to be expected.

The 538 patients (72 % of survivors) where both GCS at ICU discharge and GOS at 12 months after trauma had been recorded were included in this analysis. Of these, 57 % had a GCS score of 13–15 at ICU discharge, 19 % had a score of 10–12, 9 % had a score of 7–9 and 15 % had a score of 3–6.

The data on age, gender, trauma mechanisms, and severity of trauma and TBI, respectively, are given in Table 1. There were some significant differences between the groups. Patients with higher GCS scores at ICU discharge were younger, more likely to be injured during road traffic accidents, had lower ISS, had lower AIS scores for the region “head”, had higher GCS scores at admission, had a higher rate of normal pupils and had lower rates of closed basal cisterns and significant midline shift, respectively, on the first CT scan. The probability of survival increased with increasing GCS scores.

**Table 1:** Age, gender and trauma severity

GCS score at ICU discharge	3–6	7–9	10–12	13–15	Total	*p*-Value
Patients						
(*n*)	83	46	101	308	538	
(%)	15.4	8.6	18.8	57.2	100.0	
Age (years, mean)	49.3	43.7	39.2	37.6	40.2	<0.001
Age (years, SD)	19.4	22.4	22.1	19.5	20.6	
Gender male (% of patients)	69.9	65.2	74.3	76.0	73.8	n.s.
Alcohol intoxication (% of patients)	24.3	37.0	24.2	28.8	28.1	n.s.
Trauma mechanism (% of patients)						
Blunt assault	1.2	4.3	5.0	5.2	4.5	n.s.
Gunshot	1.2	0.0	3.0	2.3	2.0	n.s.
Fall <3 m	39.8	37.0	20.8	22.7	26.2	<0.001
Fall >3 m	3.6	10.9	8.9	5.5	6.3	n.s.
Fall total	43.4	47.8	29.7	28.2	32.5	<0.01
Bicycle	6.0	8.7	5.0	5.2	5.6	n.s.
RTA: motorcycle	4.8	2.2	9.9	9.7	8.4	n.s.
RTA: car driver	18.1	10.9	17.8	14.9	15.6	n.s.
RTA: car passenger	4.8	0.0	7.9	9.7	7.8	n.s.
RTA: pedestrian	13.3	4.3	10.9	11.7	11.2	n.s.
RTA total	41.0	17.4	46.5	46.1	42.9	<0.05
Other	3.6	13.0	8.9	9.4	8.7	n.s.
Unknown	3.6	8.7	2.0	3.6	3.7	n.s.
Severity of trauma						
ISS (median)	26	27.5	27.0	24.0	25.0	<0.001
ISS (IQR)	16–34.5	17–33.75	18–34	16–29	16–33	
Concomitant injury with AIS >2 (% of patients)						
None	53.0	58.7	45.5	53.2	52.2	n.s.
To 1 body region	26.5	21.7	21.8	24.0	23.8	n.s.
To 2 body regions	13.3	10.9	26.7	14.3	16.2	<0.05
To >2 body regions	7.2	8.7	5.9	8.4	7.8	n.s.
Severity of TBI						
First GCS score (median)	4.0	5.0	6.0	7.0	7.0	<0.001
First GCS score (IQR)	3–6	3–7	4–8	6–9	4–8	
AIS head score (median)	4.0	4.0	4.0	4.0	4.0	<0.001
AIS head score (IQR)	4–5	4–5	3–4	3–4	3–4	
Basal cisterns closed (% of patients)	24.1	6.7	7.5	3.1	7.8	<0.001
Midline shift <5 mm (% of patients)	54.9	48.8	62.4	72.8	65.5	<0.001
Normal pupils (% of patients)	43.9	61.0	60.4	69.9	63.3	<0.001
Probability of survival (median)	60.0	79.0	82.0	90.0	85.0	<0.001
Probability of survival (IQR)	38.0–80	52.5–87	62.5–92	79.0–96	65.0–95	

*GCS* Glasgow Coma Scale, *ICU* intensive care unit, *RTA* road traffic accident, *ISS* Injury Severity Score, *IQR* interquartile range, *AIS* Abbreviated Injury Scale

Data on lesions, treatment and outcomes are given in Table 2. With regard to predominant lesions on the first CT scan, the rates of epidural haematoma increased and the rates of subdural haematoma decreased with increasing GCS scores. The rates of pre-hospital airway management (i.e. endotracheal intubation or insertion of a laryngeal mask airway) and intracranial pressure (ICP) monitoring decreased significantly with increasing GCS scores. Almost half of the patients of the group with the highest GCS scores did not need neurosurgery. The median duration of ICU stay was 10 (5–21) days, without relevant differences between the groups. Patients with higher GCS scores at ICU discharge had a shorter hospital stay. One-year outcomes showed a significant correlation to the GCS scores at ICU discharge. Patients with GCS scores 13–15 had a favourable outcome in 93 % of patients, and this rate decreased to 83, 37 and 10 %, respectively, in the groups with lower GCS scores. The mortality rate was 45 % in the group with GCS scores 3–6 and decreased to 24, 5 and 3 %, respectively, with increasing GCS scores. Forty of the 62 patients (65 %) who died after ICU discharge died prior to hospital discharge; the main causes were cardio-vascular problems, respiratory problems or pulmonary embolism; “brain death” was given as the cause of death in seven cases with GCS scores 3–6. The cause of death was not available in the 22 cases who died at home (*n* = 5), during rehabilitation (*n* = 11) or at a care centre (*n* = 6).

**Table 2:** Lesions, treatments and outcomes

GCS score at ICU discharge	3–6	7–9	10–12	13–15	Total	*p*-Value
Patients (*n*)	83	46	101	308	538	
Predominant lesion (% of patients)						
Normal CT scan	3.6	0.0	2.0	6.2	4.5	n.s.
Diffuse oedema	3.6	4.3	3.0	2.9	3.2	n.s.
Subarachnoid haemorrhage	3.6	4.3	5.0	6.2	5.4	n.s.
Contusion	15.7	26.1	15.8	22.1	20.3	n.s.
Epidural haematoma	7.2	13.0	21.8	20.1	17.8	<0.05
Subdural haematoma	49.4	45.7	42.6	29.5	36.4	<0.01
Intracerebral haemorrhage	15.7	6.5	9.9	11.7	11.5	n.s.
Not specified	1.2	0.0	0.0	1.3	0.9	n.s.
Treatment						
Pre-hospital airway management (% of patients)	77.1	63.0	65.3	55.8	61.5	<0.01
Helicopter transport (% of patients)	25.3	15.2	15.8	20.1	19.7	n.s.
Direct transfer (% of patients)	69.9	69.6	67.3	68.2	68.4	n.s.
ICP monitoring (% of patients)	67.5	69.6	53.5	40.3	49.4	<0.001
Neurosurgery (% of patients)	72.3	78.3	72.3	54.2	62.5	<0.001
Ventilation days (median)	10.0	11.0	7.0	5.0	7.0	<0.001
Ventilation days (IQR)	7.0–12	5.3–20.3	3.0–13	2.0–13	3.0–14	
ICU stay (days, median)	10.0	14.0	10.0	10.0	10.0	<0.001
ICU stay (days, IQR)	9.5–19	10–25.5	5–20.5	4.0–22	5.0–21	
Hospital stay (days, median)	27.9	28.4	21.8	18.5	21.6	<0.001
Hospital stay (days, IQR)	16.9–43	19.4–54.3	15.4–35.6	10.7–34.2	12.4–38.9	
One-year outcome (% of patients)						
Good recovery	3.6	15.2	45.5	70.1	50.6	<0.001
Moderate disability	6.0	21.7	37.6	23.1	23.0	<0.001
Severe disability	20.5	15.2	11.9	3.9	8.9	<0.001
Persistent vegetative	25.3	23.9	0.0	0.0	5.9	<0.001
Death	44.6	23.9	5.0	2.9	11.5	<0.001
Favourable outcome	9.6	37.0	83.2	93.2	73.6	<0.001

*GCS* Glasgow Coma Scale, *ICU* intensive care unit, *ICP* intracranial pressure, *IQR* interquartile range

Factors that may have contributed to long-term outcomes are listed in Table 3. There was no significant “centre effect”. In the patients with favourable outcome, significant differences were found for ISS, AIS scores, rates of neurosurgery (all lower with better GCS at ICU discharge) and first GCS score. In the patients with unfavourable outcome, significant differences were found for the first GCS score (higher with better GCS score at ICU discharge) and rate of ICP monitoring (lower with better GCS at ICU discharge). Age and first GCS score were the only factors that revealed significant differences between patients with favourable and unfavourable outcome.

**Table 3:** Factors influencing long-term outcome (12-month outcome)

GCS score at ICU discharge	3–6	7–9	10–12	13–15	Total	*p*-Value
Patients (*n*)						
Total	83	46	101	308	538	
Favourable	8	15	84	287	394	
Unfavourable	75	31	17	21	144	
Patients (%)						
Favourable	9.6	32.6	83.2	93.2	73.2	<0.001
Age (years, mean)						
Favourable	44.4	33.9*	36.0**	36.3***	36.3	n.s.
Unfavourable	49.8	48.4*	55.4**	55.0***	50.9	n.s.
ISS (median)						
Favourable	26.0	26.0	27.0	22.0	25.0	<0.001
IQR	22.75–30	11.0–33	21.0–34	16.0–29	17.0–33	
Unfavourable	26.0	29.0	16.0	26.0	26.0	n.s.
IQR	16.0–35	16.0–35	16.0–25	20.0–34	16.0–34	
First GCS score (median)						
Favourable	4.0	7.0*	6.0	7.0	7.0	<0.001
IQR	4–4.5	5.5–7	4.0–8	6.0–9	5.0–9	
Unfavourable	4.0	4.0*	6.0	7.0	5.0	<0.001
IQR	3.0–6	3.0–6	4.0–8	7.0–8	3–7.25	
AIS head score (median)						
Favourable	4.0	4.0	4.0	4.0	4.0	<0.05
IQR	4.0–5	4.0–5	3.0–4	3.0–4	3.0–4	
Unfavourable	4.0	4.0	4.0	4.0	4.0	n.s.
IQR	4.0–5	4.0–5	4.0–4	4.0–4	4.0–5	
Normal pupils (% of patients)						
Favourable	50.0	54.5	58.2	70.8	67.2	n.s.
Unfavourable	43.2	63.3	70.6	57.9	52.9	n.s.
BC closed (% of patients)						
Favourable	0.0	0.0	7.9	2.5	3.6	n.s.
Unfavourable	26.8	9.7	5.9	11.1	18.2	n.s.
ML shift <5 mm (% of patients)						
Favourable	75.0	61.5	61.8	73.6	70.5	n.s.
Unfavourable	52.7	43.3	64.7	61.1	53.2	n.s.
ICP monitoring (% of patients)						
Favourable	37.5	60.0	47.6	40.4	42.6	n.s.
Unfavourable	70.7	74.2	82.4	38.1	68.1	<0.05
Neurosurgery (% of patients)						
Favourable	50.0	66.7	70.2	54.0	57.9	<0.05
Unfavourable	74.7	83.9	82.4	57.1	75.0	n.s.

Bold font indicates significant differences between favourable and unfavourable patients (*<0.05; **<0.01; ***<0.001)

*GCS* Glasgow Coma Scale, *ICU* intensive care unit, *ISS* Injury Severity Score, *AIS* Abbreviated Injury Scale, *IQR* interquartile range, *BC* basal cisterns, *ML* midline, *ICP* intracranial pressure

The multivariate analysis (Table 4) revealed that age (per year increase) and GCS score at ICU discharge were the only factors that influenced outcomes in each of the GCS groups; all other covariates (ISS, AIS score, pupils, basal cisterns, midline shift) had no significant effect. This is due to the fact that all these factors had significant influence upon the GCS score at ICU discharge.

**Table 4:** Factors influencing long-term outcome and post-ICU mortality (reference category = GCS group 3–6). Only significant factors are listed

	Odds ratio	CI low	CI high	*p*-Value
Long-term outcome (favourable = 1)				
GCS 7–9	3.99	1.48	10.82	<0.001
GCS 10–12	48.82	19.04	125.25	<0.001
GCS 13–15	129.65	53.32	315.46	<0.001
Age	0.96	0.95	0.98	<0.001
Post-ICU mortality (survived = 1)				
GCS 7–9	2.41	1.01	5.77	<0.05
GCS 10–12	14.74	5.17	41.99	<0.001
GCS 13–15	22.11	9.71	50.33	<0.001
Age	0.95	0.94	0.97	<0.001

*CI* confidence interval, *GCS* Glasgow Coma Scale, *ICU* intensive care unit

## Discussion

It is well known that the GCS score at hospital admission has prognostic value [16], and it is an important factor in all prognostic scores [17]. The most detailed analysis of the effects of GCS scores on outcomes after severe TBI was done in the IMPACT study [16]. The investigators found that the GCS score at hospital admission was strongly related to the GOS score at 6 months after trauma [odds ratio (OR) 1.7–7.5]. It is also well known that the GOS score at 3 months after trauma may be used to predict long-term outcomes [11]. The prognostic value of the GCS score at ICU discharge has not been investigated so far. As expected, we found a clear association between GCS scores at ICU discharge and long-term outcomes. Only two factors have significant influences upon long-term outcomes as well as post-ICU mortality: “age” and “GCS score after ICU discharge”.

Our study confirms some results from other studies. Berardino et al. [18] investigated the relationship between clinical status and treatment intensity in the ICU and the occurrence of life-threatening complications after ICU discharge in 39 patients with brain injuries. They found that the factors “age >50 years” and “GCS score <6” were associated with a ten-fold and seven-fold risk, respectively, of complications after ICU discharge. Low GCS scores as well as higher age were associated with a higher rate of unfavourable outcome in our study, too.

Regarding factors that influence outcomes, our study also confirms results from earlier studies. The most important factor, “age”, has been studied in detail by Hukkelhoven et al. [19]. In the largest study carried out so far, they found that the odds for poor outcome increased by 40–50 % per 10 years of age. One or both unreactive pupils were significantly associated with poor outcome (OR 2.71–7.31) in the study by Marmarou et al. [16]; in our study, lower rates of normal pupils were associated with lower GCS scores at ICU discharge. With regard to CT findings, in our study, patients with higher GCS scores had higher rates of epidural haematoma and lower rates of subdural haematoma. Maas et al. [20] demonstrated that the odds for unfavourable outcome were lower for patients with epidural haematoma (OR 0.64) and were higher for patients with subdural haematoma (OR 2.14); our data seem to confirm this.

### Limitations of this study

The main limitation is that “discharge from the ICU” is not a fixed point in time. The timing of the discharge from the ICU might actually depend on the GCS score of the patients, and if a previously comatose patient shows improvement, discharge may be postponed for a couple of days. In our study, the mean ICU stay was not different between the groups but the ranges were wide; the GCS scores used for this analysis were taken somewhere between 5 and 60 days after trauma. This was partly due to the fact that nearly half of the patients had one or more additional significantly injured body regions. However, there is a similar problem with “discharge from the hospital”; this is not a fixed point in time either, yet, “hospital outcome” is used as an endpoint in many studies. Differences regarding TIL and quality of care between study centres might be considered as another serious limitation. There is no question that these differences lead to differences in mortality [1] but we were unable to find any significant effects upon post-ICU mortality. Thus, the course of recovery of patients from a centre with low TIL and/or poor treatment quality is comparable to that of patients from centres with high TIL and/or good treatment quality once they have survived the ICU phase.

What are the possible implications of our study? Firstly, we confirmed the assumption that the GCS score at ICU discharge is related to long-term outcome, and we confirmed that age and trauma severity are important factors, too. Secondly, our data could be used to provide evidence-based probabilities of outcomes to relatives of patients with severe TBI; this might be useful for family counselling at ICU discharge of a patient. For example, patients with an age >60 years and low GCS scores at ICU discharge are very unlikely to achieve a “good outcome”, especially if they had had neurosurgery during the course of their treatment. Thirdly, the GCS score at ICU discharge—maybe in combination with age—might be useful as a surrogate parameter for long-term outcome if our findings are confirmed by further studies.

## Conclusion

We conclude that the Glasgow Coma Scale (GCS) score at intensive care unit (ICU) discharge predicts long-term outcome at 12 months after severe traumatic brain injury (TBI). “Age” is the most important factor influencing the GCS score at ICU discharge, as well as long-term outcome.
